# Adiposity and Blood Pressure in Children and Adolescents: A National Cross-Sectional Study in China

**DOI:** 10.3390/healthcare14183059

**Published:** 2026-09-17

**Authors:** Liang Wang, Shutong Yang, Youjia Li, Ling Zhang, Weixin Chen, Jiaming Yan, Wenfei Zhu, Lin Zhai, Yuliang Sun

**Affiliations:** 1 School of Physical Education, Shaanxi Normal University, Xi’an 710119, China; lwslps@snnu.edu.cn (L.W.); yst1023371019@163.com (S.Y.); 7lyj@snnu.edu.cn (Y.L.); zhangling1@snnu.edu.cn (L.Z.); chewxin@snnu.edu.cn (W.C.); yjm1511094466@snnu.edu.cn (J.Y.); wzhu@snnu.edu.cn (W.Z.); 2 College of Arts and Sports, Dong-A University, Busan 49315, Republic of Korea; 3 School of Physical Education and Sport Training, Xi’an Physical Education University, Xi’an 710068, China

**Keywords:** adiposity, blood pressure, screening-defined hypertension, body mass index, waist-to-height ratio, children, adolescents

## Abstract

**Background:** Higher blood pressure (BP) in childhood is an important predictor of cardiovascular disease later in life, and adiposity is an important modifiable factor associated with BP. However, updated national evidence on the associations of general and central adiposity indicators with BP outcomes among Chinese children and adolescents remains limited. **Methods:** This cross-sectional study was based on physical health data collected from 146,399 children and adolescents across 30 provinces in China between October and November 2019. Height, weight, waist circumference, and BP were measured using standardized procedures, and adiposity was assessed using body mass index (BMI) and waist-to-height ratio (WHtR). Linear mixed-effects models were used to evaluate associations of BMI and WHtR with continuous BP outcomes, and generalized linear mixed-effects models were used for screening-defined hypertension (SDH), adjusting for sociodemographic, dietary, and physical activity variables. **Results:** Mean systolic BP and diastolic BP were 107.40 (SD 12.65) mmHg and 67.78 (SD 9.66) mmHg, respectively. After multivariable adjustment, each 1-unit increase in BMI was associated with increased odds of SDH (OR = 1.131, 95% CI: 1.123, 1.139), and each 0.1-unit increase in WHtR was associated with increased odds of SDH (OR = 1.798, 95% CI: 1.732, 1.866). **Conclusions:** Higher BMI and WHtR were associated with higher BP levels and greater odds of SDH among Chinese children and adolescents. These findings highlight the importance of early identification and prevention of adiposity-related BP abnormalities in youth.

## 1. Introduction

Blood pressure (BP) in children and adolescents is an important early indicator of cardiovascular health [1]. Elevated BP during childhood and adolescence tends to track into adulthood and is associated with higher odds of later hypertension and cardiovascular disease [1,2,3,4]. In addition, hypertensive children may already show evidence of target organ damage, such as early atherosclerosis, left ventricular hypertrophy, and increased carotid intima-media thickness [5,6]. However, childhood hypertension remains under-recognized, partly because of limited awareness and the complexity of pediatric BP standards [7]. Therefore, identifying modifiable contributors to elevated BP during childhood and adolescence is important for early cardiovascular disease prevention.

Overweight and obesity are widely recognized as major modifiable factors associated with elevated BP in children and adolescents [8,9,10]. In particular, central adiposity and excess visceral fat accumulation have been closely linked to adverse cardiometabolic profiles, including elevated BP [11]. Globally, the burden of pediatric obesity has risen markedly, with an estimated 50 million girls and 74 million boys classified as obese in 2016 [12]. Alongside the obesity epidemic [13], increases in systolic BP (SBP) and diastolic BP (DBP) have been reported in children [14], contributing to a high and rising prevalence of hypertension among children and adolescents worldwide [15,16]. A similar pattern has been observed in China, where national data showed a marked increase in the prevalence of hypertension among children and adolescents from 1991 to 2004 [17].

Among various anthropometric measures, body mass index (BMI) and waist-to-height ratio (WHtR) have emerged as simple and widely used indicators for assessing obesity-related cardiometabolic risk in children and adolescents [18,19,20]. BMI is commonly used as an indicator of general adiposity [21], whereas WHtR is an indicator of central adiposity and abdominal fat distribution [22]. Both measures have been shown to be positively associated with SBP and DBP [18,23,24]. Although previous Chinese studies have reported associations between adiposity indicators and elevated BP or hypertension in children and adolescents [25,26,27], existing evidence has often been based on earlier data, selected subgroups, or geographically limited samples. Updated national evidence is therefore needed to clarify whether general and central adiposity remain consistently associated with SBP, DBP, and hypertension in contemporary Chinese youth.

Therefore, using a recent large national sample of Chinese children and adolescents aged 9–17 years, we examined the associations of BMI and WHtR with SBP, DBP, and screening-defined hypertension (SDH). Using BMI and WHtR as complementary indicators of general and central adiposity, this study provides updated national evidence on their associations with pediatric BP outcomes in Chinese youth.

## 2. Materials and Methods

### 2.1. Study Overview and Participants

**Figure 1:**
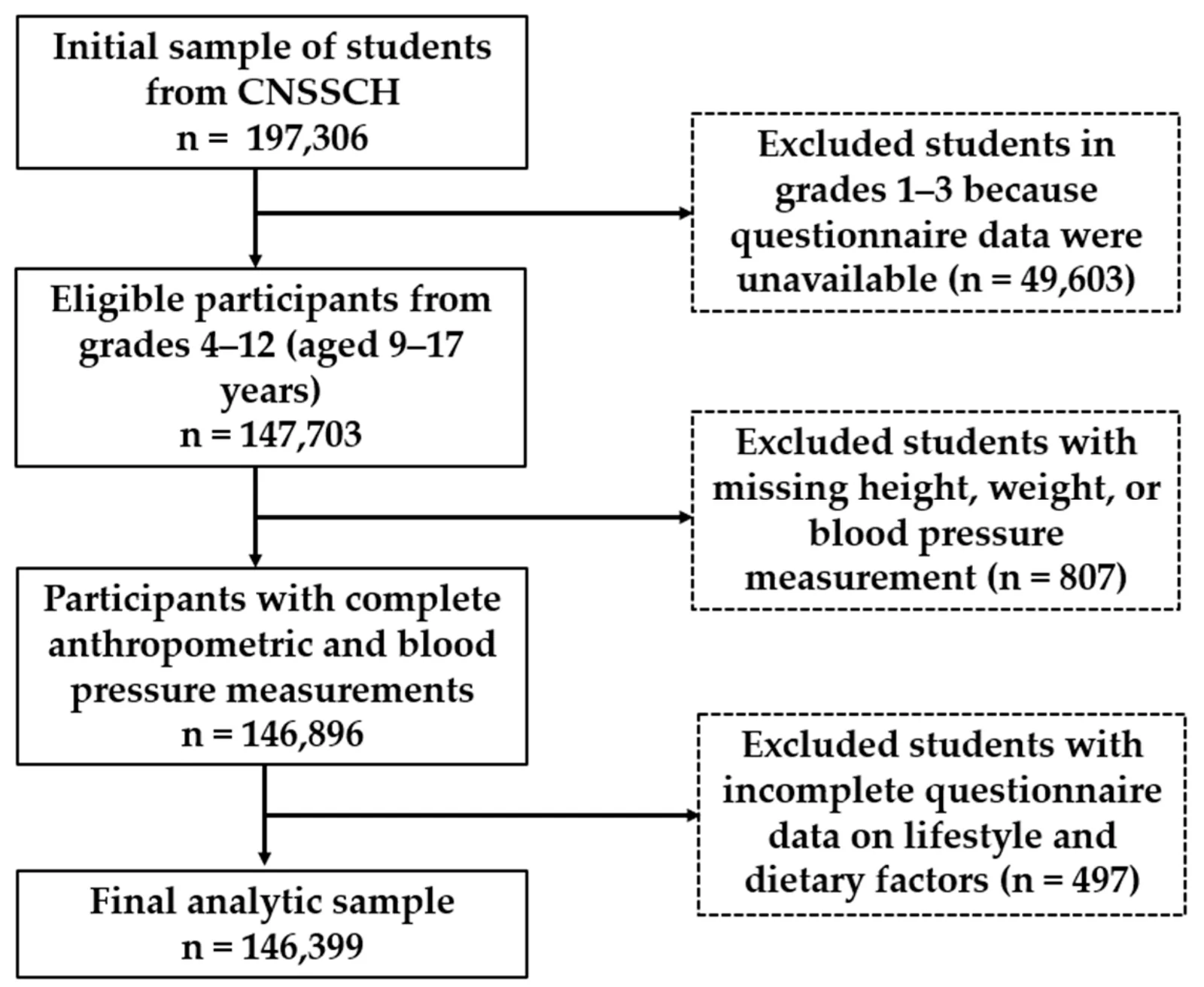
Process for screening participants.

The present study was a cross-sectional analysis based on data from the Chinese National Survey on Students’ Constitution and Health (CNSSCH), a national surveillance project designed to assess the physical fitness and health status of Chinese students. The CNSSCH is a nationwide school-based survey. Data were collected from 15 October to 15 November 2019. Students who were unable to participate in physical fitness assessments because of health conditions were excluded according to the survey protocol. A total of 197,306 students were initially sampled from the CNSSCH. The analytic population was restricted to students from the fourth grade of primary school to the third grade of high school (aged 9–17 years), because questionnaire data were unavailable for students in grades 1–3. After this restriction, 147,703 participants remained eligible for the present analysis. After further excluding students with missing anthropometric data, BP measurements, or questionnaire-based covariates, 146,399 participants were included in the final analysis (Figure 1).

The participant selection process was as follows: 30 provinces, municipalities, and autonomous regions of mainland China were included (except Hubei Province due to the COVID-19 pandemic). Cities within each province were divided into four economic tiers based on GDP [28], and one city from each tier was randomly selected as a sampling site. Within selected sites, counties and schools were randomly selected, including urban and rural primary schools, middle schools, and high schools. Students were subsequently selected through random sampling of grades and classes, and all eligible students in selected classes were invited to participate. Before the survey, school teachers explained the study procedures to the participating students and their legal guardians. Informed consent was obtained from the legal guardians of all participating students, and participation was voluntary. The study protocol was approved by the Ethics Committee of Shaanxi Normal University (201916019), and all procedures were conducted in accordance with the Declaration of Helsinki.

### 2.2. Measurement

BMI and WHtR were used as complementary anthropometric indicators of adiposity, with BMI representing general adiposity and WHtR reflecting central adiposity relative to height. (1) BMI assessment: Height and weight were measured using an electronic measuring instrument, with participants wearing light clothing and no shoes, to the nearest 0.1 cm and 0.1 kg, respectively. BMI was then calculated as weight in kilograms divided by height in meters squared (kg/m^2^). (2) WHtR assessment: Waist circumference (WC) was measured at the end of a normal exhalation using a non-elastic tape measure in a horizontal plane at the midpoint between the lower rib margin and the iliac crest. WHtR was subsequently calculated as waist circumference (in centimeters) divided by height (in centimeters).

BP was measured on the right arm by doctors from local hospitals using an automated electronic sphygmomanometer (HBP-1300, OMRON Healthcare Co., Ltd., Kyoto, Japan), which has been validated for use in Chinese children and adults [29]. An appropriately sized cuff, including pediatric cuffs for participants with smaller mid-arm circumferences, was selected based on each participant’s arm size to ensure accurate measurements in children and adolescents. Before BP measurement, participants were asked to avoid intense physical activity for 1 to 2 h and to rest in a seated position for 10 to 15 min. During measurement, participants remained seated with the right arm supported at heart level and the palm facing upward, and the cuff was placed approximately 2 cm above the elbow crease. BP was measured twice, and if the difference between the two readings exceeded 10 mmHg, a third measurement was obtained. The average of the two closest readings was used in the analysis.

Screening-defined hypertension (SDH) was identified using the BP screening thresholds recommended for Chinese children and adolescents aged 7–17 years [30]. In the present study, participants aged 9–17 years with SBP and DBP at or above the sex-, age-, and height-specific 95th percentile were classified as having SDH. All staff participating in this survey received standardized training, and all measurement instruments were strictly calibrated before use and regularly recalibrated. Measurements were conducted according to the specified procedures. Recorded data were entered twice by trained staff to ensure accuracy. Because the survey did not include clinical etiological evaluation for secondary hypertension and BP measurements were obtained during a single survey visit without repeated assessments at separate time points, clinical confirmation of hypertension was not possible. Therefore, the BP outcome was defined as SDH rather than clinically diagnosed hypertension.

### 2.3. Covariates

Covariates included in the analysis were selected based on previous studies and the availability of relevant data. Socio-demographic characteristics and selected lifestyle factors were collected using a structured questionnaire completed by the participants. The questionnaire was adapted from the 2014 National Survey on Students’ Constitution and Health developed by the Ministry of Education. Socio-demographic covariates included sex, age, ethnicity, and residence. Ethnicity was categorized as Han or non-Han, and residence was categorized as urban or rural.

Physical activity levels were assessed using items from the Health Behaviour in School-aged Children (HBSC) survey questionnaire, which has demonstrated acceptable test–retest reliability among Chinese adolescents [31]. The same instrument has been used in previous studies [32]. The time spent in moderate-to-vigorous physical activity (MVPA) was derived from self-reported data collected through this instrument. Specifically, the questionnaire included the following items: “Over the past 7 days, on how many days did you engage in light, moderate, and vigorous physical activities?” and “What was the average duration (in minutes per day) for each intensity level? Based on the reported daily time spent in MVPA, activity duration was categorized into three levels: <30 min/day, 30–60 min/day, and >1 h/day. Information on breakfast, milk, and egg intake was collected through the questions “How many days did you have breakfast in the last week?”, “How many days did you eat eggs in the last week?” and “How many days did you drink milk in the last week?” and categorized into three groups: ≤3 days/week, 4–6 days/week, or every day. Milk and egg intake were included as indicators of habitual dietary behavior and potential covariates because previous evidence has linked dietary patterns with adiposity and BP-related outcomes [33]. The selection of these dietary-related variables was also guided by their availability in the survey.

### 2.4. Statistical Analysis

All analyses were conducted using R software (version 4.4.2; R Studio) with the *lme4* and *lmerTest* packages. Linear and generalized linear mixed-effects models were fitted using the lmer() and glmer() functions, respectively. Continuous variables are presented as means and standard deviations (SDs), and categorical variables are presented as frequencies and percentages. Associations of adiposity indicators with BP outcomes were examined using three progressively adjusted mixed-effects models. In all models, adiposity indicators (BMI or WHtR) and covariates were specified as fixed effects, whereas province was included as a random intercept to account for province-level clustering among participants. Model 1 was adjusted for age and sex. Model 2 was additionally adjusted for ethnicity (Han vs. non-Han) and region (urban vs. rural). Model 3 was further adjusted for MVPA, breakfast intake, and milk and egg intake.

Linear mixed-effects models were used to evaluate associations of BMI and WHtR with continuous BP outcomes, whereas generalized linear mixed-effects models with a logit link function were used to examine SDH status. Effect estimates were presented as regression coefficients (β) for SBP and DBP and odds ratios (ORs) with 95% confidence intervals (CIs) for SDH. Associations were expressed per 1-unit increase in BMI and per 0.1-unit increase in WHtR. Pearson correlation coefficients were calculated to assess the correlation between BMI and WHtR, and variance inflation factors (VIFs) were calculated to evaluate potential multicollinearity. Additionally, mutually adjusted mixed-effects models including both BMI and WHtR simultaneously were conducted to determine whether each adiposity indicator provided independent information regarding SDH. Restricted cubic spline (RCS) analyses were conducted within the mixed-effects modeling framework to assess potential non-linear associations of BMI and WHtR with SDH. Four knots were placed at the 5th, 35th, 65th, and 95th percentiles [34]. Non-linearity was evaluated using likelihood ratio tests comparing models with linear and spline terms.

Stratified analyses were performed by sex and age group using the same mixed-effects models as the primary analyses. Participants were categorized into three age groups (9–11, 12–14, and 15–17 years). Formal interaction tests were conducted by including interaction terms between each adiposity indicator (BMI and WHtR) and sex or age group in the corresponding mixed-effects models for SBP, DBP, and SDH. As a sensitivity analysis, BMI was classified as underweight, normal weight, overweight, or obesity according to Chinese age- and sex-specific BMI reference criteria for school-aged children and adolescents, while WHtR was categorized into sample-specific quartiles. Moreover, WHtR was additionally categorized using a previously proposed threshold of 0.46 for Asian children and adolescents [35], and the associations with SDH were re-evaluated to assess the robustness of the findings. The associations of these categorical adiposity indicators with SDH were then examined in the overall sample. A two-tailed *p* value < 0.05 was considered statistically significant.

## 3. Results

### 3.1. Participant Characteristics

**Figure 2:**
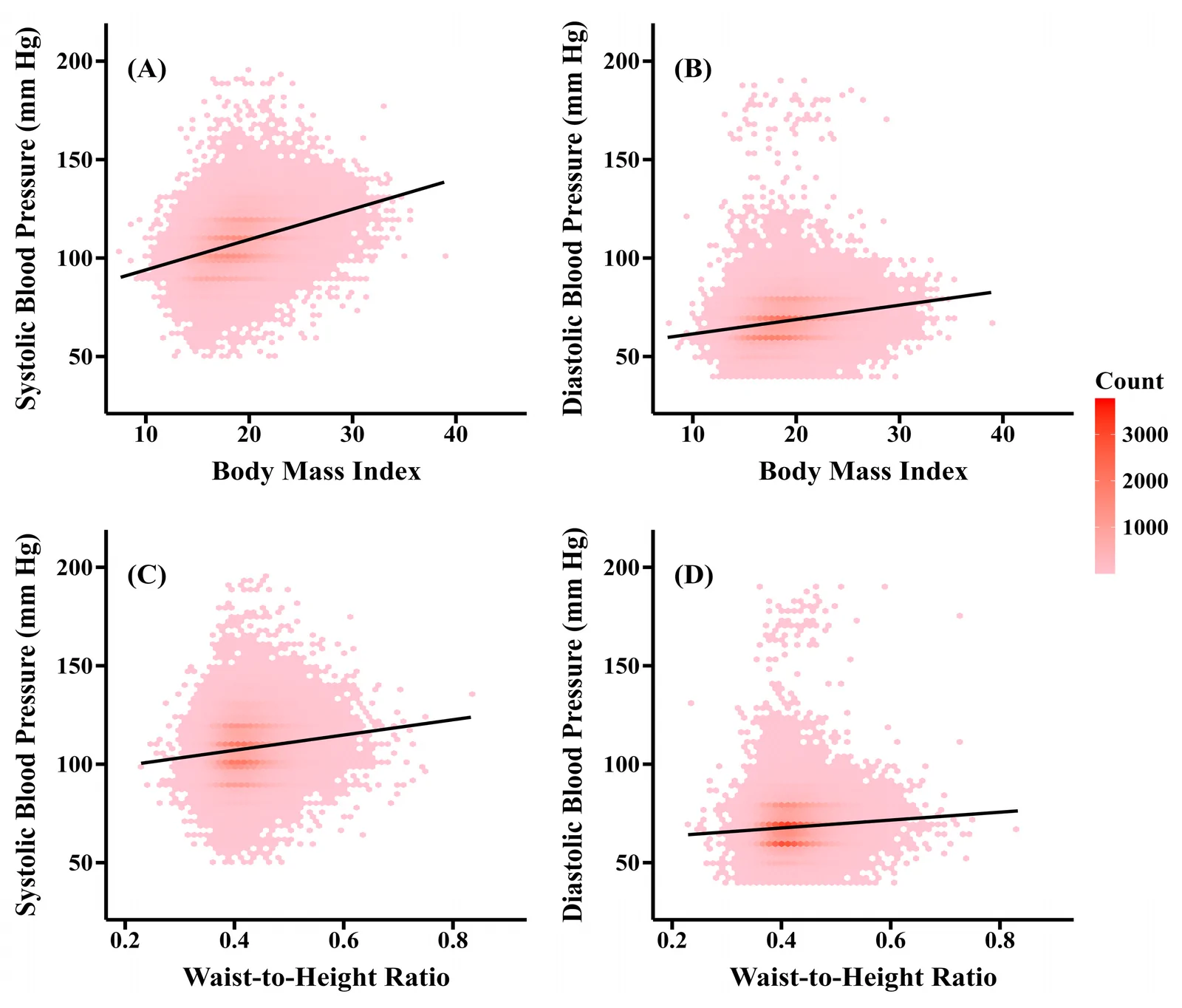
Hexbin density plots showing the associations of body mass index and waist-to-height ratio with blood pressure. (**A**) Body mass index and systolic blood pressure. (**B**) Body mass index and diastolic blood pressure. (**C**) Waist-to-height ratio and systolic blood pressure. (**D**) Waist-to-height ratio and diastolic blood pressure. Darker red indicates a higher density of observations, and the black line represents the fitted linear trend. Body mass index was calculated as weight in kilograms divided by height in meters squared; waist-to-height ratio was calculated as waist circumference in centimeters divided by height in centimeters.

The overall characteristics of the study participants are presented in Table 1. The average age was 13.39 years [SD: 2.55], and the prevalence of SDH was 11.14% (n = 16,313). The mean BMI, WHtR, SBP, and DBP were 18.85 kg/m^2^ (SD: 2.90), 0.42 (SD: 0.04), 107.40 mmHg (SD: 12.65), and 67.78 mmHg (SD: 9.66), respectively. For the whole sample, both BMI and WHtR were positively and approximately linearly associated with SBP and DBP, with stronger associations observed for SBP than for DBP (Figure 2).

### 3.2. Main Regression Analyses

The associations between adiposity indicators and BP outcomes in Chinese children and adolescents are shown in Table 2. Across all models, both BMI and WHtR were positively associated with SBP, DBP, and SDH. In the fully adjusted Model 3, each 1-unit increase in BMI was associated with higher odds of SDH (OR = 1.131, 95% CI: 1.123, 1.139), whereas each 0.1-unit increase in WHtR was associated with higher odds of SDH (OR = 1.798, 95% CI: 1.732, 1.866). The positive associations were consistent across BP outcomes and adjustment models. Sensitivity analyses excluding breakfast, milk, and egg intake from the adjustment models yielded similar results, supporting the robustness of the main findings (Supplementary Table S1).

BMI and WHtR showed a moderate correlation (r = 0.592, *p* < 0.001), while no substantial multicollinearity was observed (VIFs < 5). In mutually adjusted mixed-effects models, both BMI (OR = 1.09, 95% CI: 1.08–1.10) and WHtR (OR = 1.29, 95% CI: 1.23–1.36 per 0.1 increase) remained independently associated with SDH.

**Figure 3:**
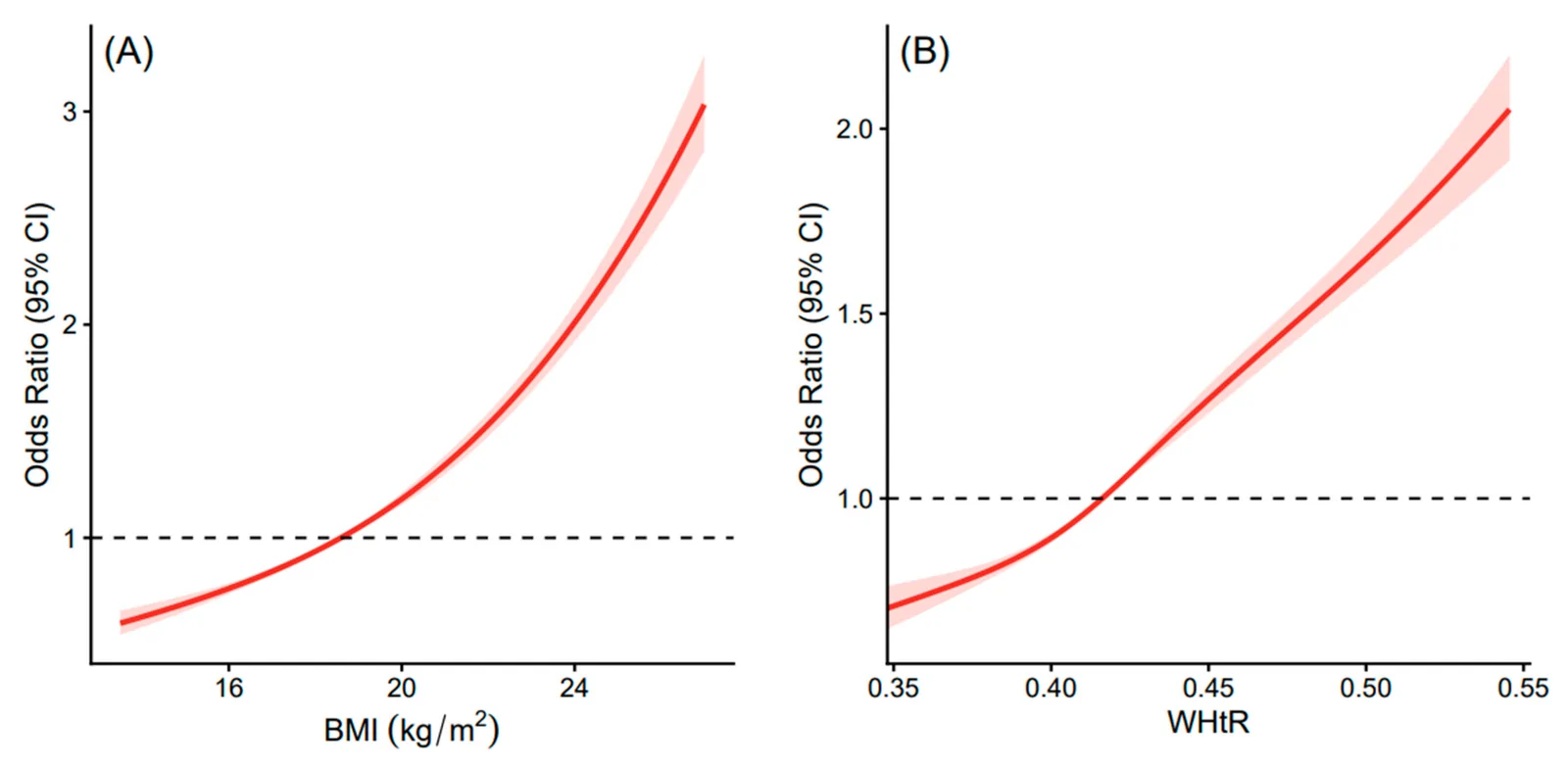
Restricted cubic spline analyses of the associations between BMI, WHtR, and SDH. (**A**) Association between BMI and SDH; (**B**) association between WHtR and SDH. Odds ratios (ORs) were calculated using the median value of each adiposity indicator as the reference. The solid line represents the estimated OR, and the shaded area represents the 95% confidence interval (CI). Abbreviations: BMI, body mass index; WHtR, waist-to-height ratio; SDH, screening-defined hypertension.

Restricted cubic spline analyses demonstrated significant overall associations of BMI and WHtR with SDH after full adjustment (*p* < 0.001). The associations were non-linear for BMI (*p* = 0.004) and WHtR (*p* = 0.014), with higher adiposity levels associated with increased odds of SDH (Figure 3).

### 3.3. Stratified and Sensitivity Analyses

Stratified analyses are presented in Table 3. In the fully adjusted model, sex significantly modified the associations of BMI with SBP and DBP (both *p* < 0.001), as well as with SDH (*p* = 0.02). Age group also significantly modified the associations of BMI with SBP, DBP, and SDH (all *p* < 0.01). For WHtR, sex significantly modified the associations with SBP (*p* < 0.001) and DBP (*p* = 0.03), whereas the interaction with SDH was not significant (*p* = 0.19). Age group also significantly modified the associations of WHtR with SBP, DBP, and SDH (all *p* < 0.001).

For BMI, sex-stratified analyses showed a stronger association with SBP in boys than in girls, whereas the associations with DBP and SDH were broadly comparable between sexes. Across age groups, the associations of BMI with SBP and DBP were strongest in the 9–11-year group and gradually attenuated in older age groups, while the association with SDH remained positive and relatively stable. For WHtR, the sex-stratified estimates were broadly similar between boys and girls, although significant sex interactions were observed for SBP and DBP. In age-stratified analyses, the associations of WHtR with SBP, DBP, and SDH were strongest in the 15–17-year group.

In sensitivity analyses, after adjustment for demographic characteristics, dietary factors, and physical activity, participants in the highest WHtR quartile had higher odds of SDH than those in the lowest quartile (OR = 1.89, 95% CI: 1.80, 1.98). Participants with WHtR ≥ 0.46 had higher odds of SDH than those with WHtR < 0.46 (OR = 1.60, 95% CI: 1.53, 1.66). Compared with the normal-weight group, participants in the BMI-defined overweight and obesity categories showed higher odds of SDH (Table 4).

## 4. Discussion

In this large nationwide sample of Chinese children and adolescents, higher BMI and WHtR were consistently associated with higher SBP, higher DBP, and greater odds of SDH. These associations were observed in both sexes and across all age groups, with age-related patterns more evident than sex-related differences. For BMI, the association with SBP was slightly stronger in boys, while the associations with DBP and SDH were similar between sexes. By age, BMI showed stronger associations with SBP and DBP in children aged 9–11 years, whereas WHtR showed the strongest associations with SBP, DBP, and SDH in adolescents aged 15–17 years.

In the current study, higher BMI and WHtR were both associated with greater odds of SDH, indicating that elevated BP in children and adolescents is associated with both general adiposity and central fat accumulation. This finding is consistent with previous pediatric studies showing that adiposity indicators, including BMI and WHtR, are positively associated with higher BP levels and increased odds of elevated BP outcomes across different populations [25,27,36,37]. In Chinese pediatric populations, BMI and WHtR have been positively associated with SBP and DBP, and both general and central obesity have been linked to higher odds of SDH [25,27]. Similar evidence has also been reported in other pediatric populations, including South Asian children and adolescents [36], supporting the broader relevance of anthropometric indicators in pediatric BP risk assessment [37]. Notably, evidence from non-overweight children aged 6–17 years suggests that adiposity-related BP risk may emerge even before conventional overweight or obesity thresholds are reached [26]. The graded associations observed across BMI and WHtR categories further suggest that the relationship between adiposity and SDH may extend across increasing levels of adiposity rather than being confined to a single obesity threshold. Although the associations of BMI and WHtR with BP outcomes were statistically significant, their clinical relevance should be interpreted in the context of the observed effect sizes, particularly given the high statistical power afforded by the large sample. These findings suggest that BMI and WHtR are practical anthropometric proxies for general and central adiposity, respectively, and may provide complementary information for identifying children and adolescents with elevated BP-related outcomes.

Several biological mechanisms may partly explain the association between adiposity and BP observed in the present study. Excess adiposity may promote renal sodium retention and volume expansion, as well as activation of the renin–angiotensin–aldosterone system and sympathetic nervous system, which together can increase vascular tone and arterial pressure [11,38,39]. Greater adiposity may also increase circulatory demand and cardiac workload, contributing to higher cardiac output and BP [40]. In addition, adipose-derived hormones, particularly leptin, may influence sympathetic activity and cardiovascular regulation, thereby contributing to obesity-related BP elevation [41]. Central adiposity may be particularly relevant in children and adolescents because visceral adipose tissue is metabolically active and may promote insulin resistance and pro-inflammatory adipokine release, while inflammation, oxidative stress, and endothelial dysfunction may further contribute to BP elevation [42,43]. The observation that weight reduction is accompanied by decreases in BP among individuals with obesity further supports the biological link between excess adiposity and BP elevation [11].

The associations between adiposity indicators and BP outcomes were generally consistent in boys and girls. Although the associations with SBP, particularly for BMI, appeared slightly stronger in boys, the sex-specific patterns for DBP and SDH were largely similar, suggesting that adiposity indicators were associated with BP outcomes in both sexes. This pattern is partly consistent with previous pediatric evidence suggesting that the relationship between fat distribution and BP may be more pronounced in boys, especially for central or trunk adiposity [44]. However, prior studies have not reported fully consistent sex-specific patterns. Some studies have shown broadly similar associations between overweight or BMI and elevated BP in boys and girls [45], whereas others suggest that body composition and pubertal maturation may influence BP differently between boys and girls across developmental stages [46,47]. For example, recent evidence indicates that cardiometabolic profiles, including BP, differ according to sex and pubertal status among children and adolescents with obesity, highlighting the potential role of biological maturation in shaping sex-specific cardiometabolic risk [46]. These discrepancies may reflect differences in age range, pubertal development, adiposity measures, fat distribution, cardiorespiratory fitness, and study populations.

Age-stratified analyses showed a clearer pattern than sex-stratified analyses. BMI showed stronger associations with SBP and DBP in children aged 9–11 years, whereas WHtR showed stronger associations with SBP, DBP, and SDH in adolescents aged 15–17 years. These findings suggest that general and central adiposity indicators may be differentially associated with BP outcomes across age groups. This interpretation is broadly consistent with previous Chinese studies showing age-related differences in high BP phenotypes and their associations with BMI [48], as well as evidence from the China Health and Nutrition Survey indicating that older adolescents, general obesity, and central obesity were positively associated with SDH [49]. One possible explanation is that BMI may more directly reflect overall body size and general adiposity in younger children, whereas WHtR may become more informative in older adolescents because it captures central fat accumulation. During puberty, rapid growth and marked changes in body composition and regional fat distribution occur, which may modify the relationship between adiposity and BP [50]. However, chronological age is only an indirect marker of biological maturation. Pubertal development may influence body composition, fat distribution, and blood pressure regulation, potentially contributing to the observed age-related differences in adiposity–BP associations [51]. Moreover, abdominal obesity has been linked to insulin resistance, metabolic abnormalities, and elevated cardiometabolic risk in children and adolescents, suggesting that central adiposity may become increasingly relevant to BP-related risk during adolescence [52,53]. Therefore, the stronger associations observed for WHtR in the 15–17-year group may partly reflect the stronger association between central fat accumulation and BP abnormalities among older adolescents. However, because pubertal stage was not directly assessed in the present study, this interpretation remains tentative. Future longitudinal studies incorporating direct assessments of pubertal maturation, such as Tanner staging, are needed to further examine this hypothesis.

From a public health perspective, these findings highlight the need for early identification and intervention among children and adolescents with adiposity-related BP abnormalities. Regular BP monitoring and follow-up assessment may be warranted in youth with elevated BMI or WHtR. Lifestyle-based interventions, including weight management, healthier dietary patterns, reduced sodium intake, increased physical activity, and less sedentary behavior, should remain the first-line strategy. In particular, schools, families, and communities should work together to create supportive environments that promote active living and healthy eating, which may contribute to the prevention and management of SDH in pediatric populations.

This study has several limitations. First, due to the cross-sectional design, causal relationships between adiposity and BP outcomes cannot be inferred. Additionally, the exclusion of students without questionnaire data or those unable to complete assessments due to health conditions may have introduced potential selection bias. Second, adiposity was assessed using BMI and WHtR only. Although these indicators are practical for large-scale epidemiological studies, they cannot directly assess body composition, fat mass, or visceral fat distribution. Third, although several demographic, lifestyle, and dietary covariates were adjusted for, residual confounding cannot be excluded because information on pubertal stage, family history of SDH, socioeconomic status, sleep duration, and sedentary behavior was unavailable. In particular, the dietary variables available in this study were relatively limited and did not include sodium and total energy intake, which may have limited our ability to fully account for nutritional confounding. Finally, the absence of Tanner staging limited our ability to further evaluate whether biological maturation contributed to the observed age- and sex-related differences in adiposity–BP associations. Future longitudinal studies with more precise adiposity assessments and more comprehensive covariate information are warranted to confirm these findings.

## 5. Conclusions

In this large national sample of Chinese children and adolescents, higher BMI and WHtR were consistently associated with higher SBP, higher DBP, and greater odds of SDH. These associations were observed in both sexes and across all age groups, with age-stratified analyses suggesting stronger BMI-related associations in children and stronger WHtR-related associations in adolescents. Although BMI and WHtR are not diagnostic tools, their assessment together with BP monitoring may provide complementary information for identifying children and adolescents with elevated BP levels in school-based and public health settings.

## Figures and Tables

**Table 1:** General characteristics of study participants.

Variables	Total (*N* = 146,399)	Non-SDH	SDH
Age (year)	13.39 ± 2.55	13.38 ± 2.55	13.52 ± 2.54
Body-mass index (kg/m^2^)	18.85 ± 2.90	18.77 ± 2.86	19.53 ± 3.18
Waist-to-Height Ratio	0.42 ± 0.04	0.42 ± 0.04	0.43 ± 0.05
Systolic blood pressure (mmHg)	107.40 ± 12.65	105.42 ± 10.97	123.20 ± 13.99
Diastolic blood pressure (mmHg)	67.78 ± 9.66	66.11 ± 8.00	81.04 ± 11.34
Boy, n (%)	69,643 (47.6%)	61,903 (47.6%)	7740 (47.4%)
Girl, n (%)	76,756 (52.4%)	68,183 (52.4%)	8573 (52.6%)
Region, n (%)			
Urban	71,389 (48.8%)	64,001 (49.2%)	7388 (45.3%)
Rural	75,010 (51.2%)	66,085 (50.8%)	8925 (54.7%)
Ethnicity, n (%)			
Han	125,804 (85.9%)	112,420 (86.4%)	13,384 (82.0%)
Non-Han	20,595 (14.1%)	17,666 (13.6%)	2929 (18.0%)
Moderate-to-vigorous physical activity, n (%)			
<30 min/day	55,742 (38.1%)	49,381 (38.0%)	6361 (39.0%)
30–60 min/day	45,642 (31.2%)	40,480 (31.1%)	5162 (31.6%)
>1 h/day	45,015 (30.7%)	40,225 (30.9%)	4790 (29.4%)
Breakfast intake, n (%)			
≤3 days/week	14,945 (10.2%)	13,047 (10.0%)	1898 (11.6%)
4–6 days/week	27,304 (18.7%)	24,444 (18.8%)	2860 (17.5%)
Daily	104,150 (71.1%)	92,595 (71.2%)	11,555 (70.8%)
Egg intake, n (%)			
≤3 days/week	90,618 (61.9%)	80,266 (61.7%)	10,352 (63.5%)
4–6 days/week	31,841 (21.7%)	28,405 (21.8%)	3436 (21.1%)
Daily	23,940 (16.4%)	21,415 (16.5%)	2525 (15.5%)
Milk intake, n (%)			
≤3 days/week	63,062 (43.1%)	55,817 (42.9%)	7245 (44.4%)
4–6 days/week	38,607 (26.4%)	34,323 (26.4%)	4284 (26.3%)
Daily	44,730 (30.6%)	39,946 (30.7%)	4784 (29.3%)

Data are presented as mean ± SD for continuous variables and n (%) for categorical variables.

**Table 2:** Associations between adiposity and BP outcomes (*N* = 146,399).

	BMI	WHtR
SBP (mmHg)	β (95% CI)	β (95% CI)
Model 1	0.873 (0.848, 0.897)	2.523 (2.385, 2.660)
Model 2	0.878 (0.853, 0.903)	2.520 (2.383, 2.658)
Model 3	0.879 (0.854, 0.903)	2.526 (2.389, 2.664)
DBP (mmHg)	β (95% CI)	β (95% CI)
Model 1	0.346 (0.326, 0.366)	1.033 (0.921, 1.145)
Model 2	0.348 (0.327, 0.368)	1.043 (0.931, 1.155)
Model 3	0.348 (0.328, 0.368)	1.044 (0.933, 1.156)
SDH	OR (95% CI)	OR (95% CI)
Model 1	1.129 (1.121, 1.136)	1.787 (1.722, 1.854)
Model 2	1.130 (1.123, 1.138)	1.795 (1.729, 1.863)
Model 3	1.131 (1.123, 1.139)	1.798 (1.732, 1.866)

Note: Estimated effects are calculated for each 1-unit increase in BMI and each 0.1-unit increase in WHtR. Model 1 was adjusted for age and sex. Model 2 was further adjusted for ethnicity and residence. Model 3 was additionally adjusted for MVPA, breakfast intake, egg intake, and milk intake. Abbreviations: BMI, body mass index; WHtR, waist-to-height ratio; BP, blood pressure; SBP, systolic blood pressure; DBP, diastolic blood pressure; SDH, screening-defined hypertension; CI, confidence interval; OR, odds ratio; β, regression coefficients.

**Table 3:** Stratified associations of adiposity indicators with blood pressure outcomes.

Outcome	Exposure	Stratification factor	Level	Effect (95% CI)
SBP (mmHg)	BMI	Sex	Boys	0.97 (0.93, 1.00)
			Girls	0.83 (0.80, 0.86)
DBP (mmHg)	BMI	Sex	Boys	0.35 (0.32, 0.38)
			Girls	0.36 (0.33, 0.39)
SDH	BMI	Sex	Boys	1.13 (1.12, 1.14)
			Girls	1.13 (1.12, 1.14)
SBP (mmHg)	BMI	Age group	9–11 years	1.16 (1.11, 1.22)
			12–14 years	1.06 (1.02, 1.11)
			15–17 years	0.90 (0.86, 0.93)
DBP (mmHg)	BMI	Age group	9–11 years	0.57 (0.52, 0.62)
			12–14 years	0.41 (0.37, 0.44)
			15–17 years	0.34 (0.31, 0.37)
SDH	BMI	Age group	9–11 years	1.13 (1.11, 1.15)
			12–14 years	1.11 (1.09, 1.12)
			15–17 years	1.14 (1.13, 1.15)
SBP (mmHg)	WHtR	Sex	Boys	2.99 (2.79, 3.19)
			Girls	2.83 (2.64, 3.03)
DBP (mmHg)	WHtR	Sex	Boys	1.17 (1.00, 1.33)
			Girls	1.19 (1.04, 1.35)
SDH	WHtR	Sex	Boys	1.84 (1.75, 1.94)
			Girls	1.79 (1.70, 1.89)
SBP (mmHg)	WHtR	Age group	9–11 years	2.68 (2.40, 2.95)
			12–14 years	2.37 (2.12, 2.63)
			15–17 years	3.98 (3.77, 4.18)
DBP (mmHg)	WHtR	Age group	9–11 years	1.17 (0.93, 1.41)
			12–14 years	0.84 (0.64, 1.05)
			15–17 years	1.62 (1.46, 1.79)
SDH	WHtR	Age group	9–11 years	1.64 (1.52, 1.78)
			12–14 years	1.76 (1.64, 1.88)
			15–17 years	2.00 (1.89, 2.11)

Note: For SBP and DBP, effect estimates are regression coefficients (β) with 95% confidence intervals (CIs). For SDH, effect estimates are odds ratios (ORs) with 95% CIs. Estimated effects were calculated for each 1-unit increase in BMI and each 0.1-unit increase in WHtR. Subgroup sample sizes were identical across all stratified analyses: boys, n = 69,643; girls, n = 76,756; 9–11 years, n = 40,843; 12–14 years, n = 47,973; and 15–17 years, n = 57,583. Abbreviations: BMI, body mass index; WHtR, waist-to-height ratio; SBP, systolic blood pressure; DBP, diastolic blood pressure; SDH, screening-defined hypertension.

**Table 4:** Associations of BMI and WHtR categories with screening-defined hypertension.

	Level	Participants	Prevalence	OR (95% CI)
WHtR	<0.39	36,599	2977 (8.13%)	Ref
quartile	0.39–0.42	36,595	3423 (9.35%)	1.16 (1.10, 1.22)
	0.42–0.45	36,605	4261 (11.64%)	1.44 (1.37, 1.51)
	≥0.45	36,600	5652 (15.44%)	1.89 (1.80, 1.98)
WHtR	<0.46	121,713	12,293 (10.10%)	Ref
cut-off	≥0.46	24,686	4020 (16.28%)	1.60 (1.53, 1.66)
BMI	Normal	125,275	13,259 (10.58%)	Ref
	Underweight	6619	483 (7.30%)	0.70 (0.64, 0.77)
	Overweight	11,432	1883 (16.47%)	1.69 (1.60, 1.78)
	Obesity	3073	688 (22.39%)	2.63 (2.40, 2.88)

Note: Results are described as odds ratios and 95% confidence intervals. Abbreviations: BMI, body mass index; WHtR, waist-to-height ratio; OR, odds ratio; Ref, reference group.

## Data Availability

The data presented in this study are available upon request from the corresponding author. The data are not publicly available due to confidentiality reasons.

## Supplementary Materials

The following supporting information can be downloaded at https://www.mdpi.com/article/10.3390/healthcare14183059/s1: Table S1. Associations between adiposity and BP (*N* = 146,399).

## Abbreviations

The following abbreviations are used in this manuscript:

BMI	body mass index
WHtR	waist-to-height ratio
WC	waist circumference
SBP	systolic blood pressure
DBP	diastolic blood pressure
BP	blood pressure
SDH	screening-defined hypertension.
CI	confidence interval
OR	odds ratio
β	regression coefficient

## References

[1] Liao Y.-Y., Ma Q., Chu C., Wang Y., Zheng W.-L., Hu J.-W., Yan Y., Wang K.-K., Yuan Y., Chen C. (2020). The Predictive Value of Repeated Blood Pressure Measurements in Childhood for Cardiovascular Risk in Adults: The Hanzhong Adolescent Hypertension Study. Hypertens. Res..

[2] Hamrahian S.M., Falkner B. (2022). Approach to Hypertension in Adolescents and Young Adults. Curr. Cardiol. Rep..

[3] Urbina E.M., Khoury P.R., Bazzano L., Burns T.L., Daniels S., Dwyer T., Hu T., Jacobs D.R., Juonala M., Prineas R. (2019). Relation of Blood Pressure in Childhood to Self-Reported Hypertension in Adulthood. Hypertension.

[4] Chen X., Wang Y. (2008). Tracking of Blood Pressure from Childhood to Adulthood: A Systematic Review and Meta-Regression Analysis. Circulation.

[5] McGill H.C., McMahan C.A., Herderick E.E., Malcom G.T., Tracy R.E., Strong J.P. (2000). Origin of Atherosclerosis in Childhood and Adolescence. Am. J. Clin. Nutr..

[6] Turer C.B., Brady T.M., de Ferranti S.D. (2018). Obesity, Hypertension, and Dyslipidemia in Childhood Are Key Modifiable Antecedents of Adult Cardiovascular Disease: A Call to Action. Circulation.

[7] Bello J.K., Mohanty N., Bauer V., Rittner S.S., Rao G. (2017). Pediatric Hypertension: Provider Perspectives. Glob. Pediatr. Health.

[8] Chiolero A., Cachat F., Burnier M., Paccaud F., Bovet P. (2007). Prevalence of Hypertension in Schoolchildren Based on Repeated Measurements and Association with Overweight. J. Hypertens..

[9] Drozdz D., Alvarez-Pitti J., Wójcik M., Borghi C., Gabbianelli R., Mazur A., Herceg-Čavrak V., Lopez-Valcarcel B.G., Brzeziński M., Lurbe E. (2021). Obesity and Cardiometabolic Risk Factors: From Childhood to Adulthood. Nutrients.

[10] Lauer R.M., Clarke W.R. (1989). Childhood Risk Factors for High Adult Blood Pressure: The Muscatine Study. Pediatrics.

[11] Hall J.E., do Carmo J.M., da Silva A.A., Wang Z., Hall M.E. (2015). Obesity-Induced Hypertension Interaction of Neurohumoral and Renal Mechanisms. Circ. Res..

[12] (2017). NCD Risk Factor Collaboration (NCD-RisC) Worldwide Trends in Body-Mass Index, Underweight, Overweight, and Obesity from 1975 to 2016: A Pooled Analysis of 2416 Population-Based Measurement Studies in 128·9 Million Children, Adolescents, and Adults. Lancet.

[13] de Onis M., Blossner M., Borghi E. (2010). Global Prevalence and Trends of Overweight and Obesity among Preschool Children. Am. J. Clin. Nutr..

[14] Muntner P., He J., Cutler J.A., Wildman R.P., Whelton P.K. (2004). Trends in Blood Pressure among Children and Adolescents. JAMA-J. Am. Med. Assoc..

[15] Maldonado J., Pereira T., Fernandes R., Santos R., Carvalho M. (2011). An Approach of Hypertension Prevalence in a Sample of 5381 Portuguese Children and Adolescents. Blood Press..

[16] Martín-Espinosa N., Díez-Fernández A., Sánchez-López M., Rivero-Merino I., Lucas-De La Cruz L., Solera-Martínez M., Martínez-Vizcaíno V., Movi-Kids group (2017). Prevalence of High Blood Pressure and Association with Obesity in Spanish Schoolchildren Aged 4–6 Years Old. PLoS ONE.

[17] Liang Y.-J., Xi B., Hu Y.-H., Wang C., Liu J.-T., Yan Y.-K., Xu T., Wang R.-Q. (2011). Trends in Blood Pressure and Hypertension among Chinese Children and Adolescents: China Health and Nutrition Surveys 1991-2004. Blood Press..

[18] Choi J.R., Koh S.B., Choi E. (2018). Waist-to-Height Ratio Index for Predicting Incidences of Hypertension: The ARIRANG Study. BMC Public Health.

[19] Hu J., Chu G., Huang F., Zhou Y., Teng C., Yang H., Shen H. (2016). Relation of Body Mass Index (BMI) to the Prevalence of Hypertension in Children: A 3 Years’ School-Based Prospective Study in Suzhou, China. Int. J. Cardiol..

[20] Moosaie F., Fatemi Abhari S.M., Deravi N., Karimi Behnagh A., Esteghamati S., Dehghani Firouzabadi F., Rabizadeh S., Nakhjavani M., Esteghamati A. (2021). Waist-To-Height Ratio Is a More Accurate Tool for Predicting Hypertension Than Waist-To-Hip Circumference and BMI in Patients With Type 2 Diabetes: A Prospective Study. Front. Public Health.

[21] Javed A., Jumean M., Murad M.H., Okorodudu D., Kumar S., Somers V.K., Sochor O., Lopez-Jimenez F. (2015). Diagnostic Performance of Body Mass Index to Identify Obesity as Defined by Body Adiposity in Children and Adolescents: A Systematic Review and Meta-analysis. Pediatr. Obes..

[22] Brambilla P., Bedogni G., Heo M., Pietrobelli A. (2013). Waist Circumference-to-Height Ratio Predicts Adiposity Better than Body Mass Index in Children and Adolescents. Int. J. Obes..

[23] Brion M.A., Ness A.R., Davey Smith G., Leary S.D. (2007). Association between Body Composition and Blood Pressure in a Contemporary Cohort of 9-Year-Old Children. J. Hum. Hypertens..

[24] Droyvold W.B., Midthjell K., Nilsen T.I.L., Holmen J. (2005). Change in Body Mass Index and Its Impact on Blood Pressure: A Prospective Population Study. Int. J. Obes..

[25] Zhao Y., Wang L., Xue B., Wang Y. (2017). Associations between General and Central Obesity and Hypertension among Children: The Childhood Obesity Study in China Mega-Cities. Sci. Rep..

[26] Tian C., Xu S., Wang H., Wang W., Shen H. (2017). Blood Pressure Effects of Adiposity in Non-Overweight Children Aged 6–17 Years. Ann. Hum. Biol..

[27] Dong B., Wang Z., Wang H.-J., Ma J. (2015). Associations between Adiposity Indicators and Elevated Blood Pressure among Chinese Children and Adolescents. J. Hum. Hypertens..

[28] Notice from the Ministry of Education and Five Other Departments on Conducting the 2019 National Survey on Students’ Physical Fitness and Health, and the Review and Verification of the National Standards for Students’ Physical Fitness and Health—Government Portal of the Ministry of Education of the People’s Republic of China. http://www.moe.gov.cn/srcsite/A17/moe_943/moe_946/201907/t20190724_392121.html.

[29] Meng L., Zhao D., Pan Y., Ding W., Wei Q., Li H., Gao P., Mi J. (2016). Validation of Omron HBP-1300 Professional Blood Pressure Monitor Based on Auscultation in Children and Adults. BMC Cardiovasc. Disord..

[30] Dong Y., Ma J., Song Y., Dong B., Wang Z., Yang Z., Wang X., Prochaska J.J. (2017). National Blood Pressure Reference for Chinese Han Children and Adolescents Aged 7 to 17 Years. Hypertension.

[31] Liu Y., Wang M., Tynjälä J., Lv Y., Villberg J., Zhang Z., Kannas L. (2010). Test-Retest Reliability of Selected Items of Health Behaviour in School-Aged Children (HBSC) Survey Questionnaire in Beijing, China. BMC Med. Res. Methodol..

[32] Zhu W., Zhang L., Zhang L., Qiu L., Guo J., Li Z., Sun Y. (2022). Association of Physical Activity and Sedentary Behaviors with the Risk of Refractive Error in Chinese Urban/Rural Boys and Girls. Sustainability.

[33] McNaughton S.A., Ball K., Mishra G.D., Crawford D.A. (2008). Dietary Patterns of Adolescents and Risk of Obesity and Hypertension. J. Nutr..

[34] Austin P.C., Fang J., Lee D.S. (2022). Using Fractional Polynomials and Restricted Cubic Splines to Model Non-proportional Hazards or Time-varying Covariate Effects in the Cox Regression Model. Stat. Med..

[35] Zong X., Kelishadi R., Hong Y.M., Schwandt P., Matsha T.E., Mill J.G., Whincup P.H., Pacifico L., López-Bermejo A., Caserta C.A. (2023). Establishing International Optimal Cut-Offs of Waist-to-Height Ratio for Predicting Cardiometabolic Risk in Children and Adolescents Aged 6–18 Years. BMC Med..

[36] Fowokan A.O., Punthakee Z., Waddell C., Rosin M., Morrison K.M., Gupta M., Teo K., Rangarajan S., Lear S.A. (2019). Adiposity Measures and Their Validity in Estimating Risk of Hypertension in South Asian Children: A Cross-Sectional Study. BMJ Open.

[37] Papalia T., Greco R., Lofaro D., Monica A., Roberti R., Bonofiglio R. (2013). Anthropometric Measures Can Better Predict High Blood Pressure in Adolescents. J. Nephrol..

[38] Hall J.E., Brands M.W., Henegar J.R., Shek E.W. (1998). Abnormal Kidney Function as a Cause and a Consequence of Obesity Hypertension. Clin. Exp. Pharmacol. Physiol..

[39] Hall J.E., Brands M.W., Henegar J.R. (1999). Mechanisms of Hypertension and Kidney Disease in Obesity. Ann. N. Y Acad. Sci..

[40] Hall J.E. (2000). Pathophysiology of Obesity Hypertension. Curr. Sci. Inc..

[41] Hirose H., Saito I., Tsujioka M., Mori M., Kawabe H., Saruta T. (1998). The Obese Gene Product, Leptin: Possible Role in Obesity-Related Hypertension in Adolescents. J. Hypertens..

[42] Wirix A.J.G., Kaspers P.J., Nauta J., Chinapaw M.J.M., Kist-van Holthe J.E. (2015). Pathophysiology of Hypertension in Obese Children: A Systematic Review. Obes. Rev..

[43] Tung J.Y.L., Poon G.W.K., Du J., Wong K.K.Y. (2023). Obesity in Children and Adolescents: Overview of the Diagnosis and Management. Chronic Dis. Transl. Med..

[44] He Q., Horlick M., Fedun B., Wang J., Pierson R.N., Heshka S., Gallagher D. (2002). Trunk Fat and Blood Pressure in Children Through Puberty. Circulation.

[45] Dong B., Ma J., Wang H.J., Wang Z.Q. (2013). The Association of Overweight and Obesity with Blood Pressure among Chinese Children and Adolescents. Biomed. Environ. Sci..

[46] Gilardini L., Croci M., Cavaggioni L., Pasqualinotto L., Bertoli S. (2024). Sex Differences in Cardiometabolic Risk Factors and in Response to Lifestyle Intervention in Prepubertal and Pubertal Subjects with Obesity. Front. Pediatr..

[47] Leccia G., Marotta T., Masella M.R., Mottola G., Mitrano G., Golia F., Capitanata P., Guida L., Contaldo F., Ferrara L.A. (1999). Sex-Related Influence of Body Size and Sexual Maturation on Blood Pressure in Adolescents. Eur. J. Clin. Nutr..

[48] Yang Y., Dong B., Wang S., Dong Y., Zou Z., Fu L., Ma J. (2017). Prevalence of High Blood Pressure Subtypes and Its Associations with BMI in Chinese Children: A National Cross-Sectional Survey. BMC Public Health.

[49] Ye X., Yi Q., Shao J., Zhang Y., Zha M., Yang Q., Xia W., Ye Z., Song P. (2021). Trends in Prevalence of Hypertension and Hypertension Phenotypes among Chinese Children and Adolescents over Two Decades (1991–2015). Front. Cardiovasc. Med..

[50] Rogol A.D., Roemmich J.N., Clark P.A. (2002). Growth at Puberty. J. Adolesc. Health.

[51] Kwarteng E.A., Shank L.M., Faulkner L.M., Loch L.K., Fatima S., Gupta S., Haynes H.E., Ballenger K.L., Parker M.N., Brady S.M. (2023). Influence of Puberty on Relationships between Body Composition and Blood Pressure: A Cross-Sectional Study. Pediatr. Res..

[52] Forkert E.C.O., Rendo-Urteaga T., Nascimento-Ferreira M.V., De Moraes A.C.F., Moreno L.A., De Carvalho H.B. (2016). Abdominal Obesity and Cardiometabolic Risk in Children and Adolescents, Are We Aware of Their Relevance?. Nutrire.

[53] Kelishadi R., Mirmoghtadaee P., Najafi H., Keikha M. (2015). Systematic Review on the Association of Abdominal Obesity in Children and Adolescents with Cardio-Metabolic Risk Factors. J. Res. Med. Sci..

