# Supplementary material for: Adiposity and Blood Pressure in Children and Adolescents: A National Cross-Sectional Study in China
Source: Healthcare (Basel). 2026 Sep 17;14(18):3059. doi: 10.3390/healthcare14183059 (PMC13606509; doi:10.3390/healthcare14183059)
Supplement: Supplementary file 1 [file healthcare-14-03059-s001.zip › healthcare-4532811-supplementary.pdf]

Supplementary Material

Supplementary Table S1 Associations between adiposity and BP (*N*=146,399).

	BMI	WHtR
SBP (mmHg)	β (95% CI)	β (95% CI)
Model 1	0.873 (0.848, 0.897)	2.523 (2.385, 2.660)
Model 2	0.878 (0.853, 0.902)	2.520 (2.382, 2.657)
Model 3	0.878 (0.853, 0.903)	2.521 (2.383, 2.658)
DBP (mmHg)	β (95% CI)	β (95% CI)
Model 1	0.346 (0.325, 0.367)	1.033 (0.921, 1.145)
Model 2	0.348 (0.327, 0.368)	1.042 (0.930, 1.154)
Model 3	0.348 (0.328, 0.368)	1.043 (0.932, 1.155)
SDH	OR (95% CI)	OR (95% CI)
Model 1	1.122 (1.115, 1.130)	1.787 (1.722, 1.854)
Model 2	1.130 (1.123, 1.138)	1.794 (1.729, 1.862)
Model 3	1.130 (1.123, 1.138)	1.795 (1.729, 1.863)

Note: Estimated effects are calculated for each 1-unit increase in BMI and each 0.1-unit increase in WHtR. Model 1 was adjusted for age and sex. Model 2 was further adjusted for ethnicity, residence. Model 3 was additionally adjusted for MVPA, breakfast intake, egg intake, and milk intake. Abbreviations: BMI, body mass index; WHtR, waist-to-height ratio; SBP, systolic blood pressure; DBP, diastolic blood pressure; CI, confidence interval; OR, odds ratio; β, regression coefficients; SDH, screening-defined hypertension.
